# Medical Studies on Ancient Rome

**Published:** 1860-09

**Authors:** 


ART. II.—*Etudes Médicales sur l'Ancienne Rome.* Par JULES ROUYER, Docteur en Médecine de la Faculté de Paris. pp. 239, 8vo. Paris, 1859.

*Medical Studies on Ancient Rome.* By JULES ROUYER, M.D. Paris, 1859.

THE author of this somewhat curious volume has collected the skimmings, and, it must be said, also a good deal of the moral scum of Roman literature, and especially poetry, to show the extent to which public baths, as well as the use of cosmetics and perfumes, and of practices alike injurious to health and adverse to morals, were resorted to by the Romans in the time of the Empire. The title of "Medical Studies" is misleading. That of Curiosities of the Personal Habits and Vices of the Inhabitants of Imperial Rome, would be more appropriate. Some details are, indeed, given of the varieties of the Roman baths and of their divisions, and the modes of bathing, and a few remarks are added on mineral springs; but of the medical practice and of the physicians of ancient Rome scarcely any notice is taken. Celsus is more spoken of as the friend of Ovid than as the author *de Re Medica*. Dr. Rouyer's chief authorities on the other subjects, after baths, viz., female conjurers, philters, abortion, eunuchs, infibulation, cosmetics, perfumes, etc., are the poets, Juvenal, Martial, Ovid, Tibullus, and Propertius, with occasional references to Celsus and Pliny among the prose writers. We shall follow in his track, but in doing so we must step very warily, in order to avoid the mud-holes and offensive and corrupt matters which lie directly in the way. In expressing ourselves thus we do not accuse the author of coarseness or obscenity in his own language, but of those of the poets whom he quotes. He, for the most part, translates the original Latin verse into plain French prose; but sometimes he admits that the terms used by the poets are too gross for a literal rendering into his own tongue.

In the first chapter, the author speaks of the public baths of Rome and of the mineral waters known to the ancients. Of the former of these themes there are such ample descriptions and details in the volume of Dr. Bell on baths, noticed in this journal last year,* that we deem it needless to present more than a few detached notices from the pages of the French author. We will only indulge in the following quotation from the American writer, just named, to show the wonderful extent and luxurious appliances of the Roman baths: "Within the vast precincts of the thermæ were found temples, palestræ for the sports of running, wrestling, boxing, pitching the quoit and throwing the javelin, and extensive libraries. Architecture, sculpture, and painting exhausted their refinements on these establishments, which, for their extent, were compared to cities: incrustations, metals and marble were all employed in adorning them. Those of which the most numerous remains are still visible are the baths of Titus, Antoninus, Caracalla, and Diocletian. In the order of time, these were of subsequent erection to the thermæ of Agrippa and of Nero. Of the magnificence of the baths of Agrippa, the relation, friend, and counselor of Augustus, an idea may be formed from the circumstance of the Pantheon serving as a vestibule to them."†

If reference be made to Antonius Musa, the physician of Augustus, to show the antiquity of hydropathy, in the successful treatment of this emperor by the use of water internally and externally for a disease of the liver, we must not fail to connect this fact with the fatal results of a similar course directed by the same physician in the case of the young Mar-

* July, 1859.

† Chap. v. p. 82.

cellus, the presumptive heir to the imperial purple; and press them on the notice of the medical reader, in illustration of the danger of the empirical employment of a remedy in persons of different constitutions and powers of endurance and reaction, not to speak of the different and contrasted pathological states at the time. The transition bath, as it is called by Dr. Bell, the Russian of modern, the Lacedemonian of ancient times, was used by the Romans, as we learn from passages in Martial and Petronus. The first of these writers says: "If the practices of the Lacedemonians suit you, you may content yourself with dry vapor, and afterwards plunge into the cold Virginal or Marcian waters."* These were the names of two of the many aqueducts which supplied Rome with water. Petronus writes: "We went to the bath, and when we were in a copious sweat we passed immediately into the reservoir of cold water."†

With increasing corruption of manners, the public baths, especially when kept open during the night, became the rendezvous of the profligate. Here the courtezans and prostitutes resorted to exercise their vile calling. Among the attendants on the baths were the *tonstrices*, or female barbers, and *tractatrices*, or shampooers, nimble rubbers and kneaders of the bodies of the bathers. These persons were at first restricted to the service of the baths for women, but after a time they were permitted to render similar offices to the bathers of the other sex; and in their turn men performed these tactile movements for the female bathers. The thermal establishments of the Romans were so arranged as to allow of room and accommodations for feasts, or *commessationes*, which lasted half of the day, and sometimes were prolonged far into the night. When Petronus, in his memorable distich, says, "our bodies are corrupted by baths, wine, and love; baths, wine, and love support life,"‡ he had in his mind the debasing and enfeebling accessories of the bath, rather than simple bathing in any of its varieties.

The private baths were scarcely less in size, and were more splendidly decorated, than the public ones of ancient Rome. Pliny the Younger, in one of his letters, describes the baths of his country-seat at Laurentium, now *Torre di Paterna*, six leagues from Rome. The Roman ladies would bathe in nothing short of reservoirs made of the

---

*Ritus si placeant tibi Laconum
Contentus potes arido vapore
Cruda Virgine Marciave mergi.—Lib. vii. ep. 42.

† Itaque balneum intravimus et sudore calefacti, momento temporis ad frigidum eximus.

‡ Balnea, vina, Venus, corrumpunt corpora nostra,
Et vitam faciunt, balnea, vina, Venus.

most precious materials, such as of silver; and Seneca inveighs against the costly and luxurious embellishments of the walls and ceilings of the baths—the marbles of Egypt inlaid with those of Numidia, mosaic borders, walls laboriously stuccoed in imitation of painting, the ceilings of the chambers covered with glass, piscinas of Thracian stone, and silver pipes for conveying the water.

The strangest vagaries in thermal or balneatory practices were indulged in by some of the Roman emperors. Tiberius, after he had retired to Capreæ, used to bathe with children, who swam and performed various antics in the cistern. Heliogabalus never bathed without previously causing to be thrown into the water of the reservoir the rarest perfumes, or the essence of saffron. This embodiment of all that was luxurious, vile, and licentious used to subject his parasites to a fashion of bathing which was not ill timed in their case. He had them made fast to a wheel, which was in part below the surface of the water, and which consequently in its revolutions secured a thorough immersion for the imperial flatterers, who were complimented by their master with the title of his dear Ixions—"*Ixionios Amicos.*" Heliogabalus, after listening once to a conversation on the subject, bethought himself of a singular method, which he carried into practice, for procuring a personal knowledge of the statistics of hernia. He had an exact list of all the persons in Rome who were afflicted in this way, and having summoned them together, he bathed with them. On other occasions he brought together all the prostitutes of one of the quarters of Rome, bathed with them, acted as depilator, perfumed them, and then collected for his own personal use the cosmetic pastes of which these women had made use. Domitian had similar tastes in the selection of his bathing companions, and in performing the like offices for them. We speak of the barbarians overrunning and destroying the Roman empire as a terrible and deplorable event. Was it not rather a necessity, an agency visibly chosen by God for the restoration of mankind from the lowest condition of debased and worn-out humanity to youth and freshness? A new birth was called for, and it could only be brought about in this violent manner.

Mineral springs were known in large numbers in the time of Pliny, who gives them names and medicinal properties, real and imaginary. On this topic, the matter furnished by Dr. Rouyer is very meagre. Were it necessary, we could supply his omissions from our own large stores, especially in what relates to the thermal waters known to the Roman world; but we do not deem the present an opportune occasion. The author just named speaks of the waters of Sinessa, in Campania, which had the double virtue of removing sterility in women and of curing insanity in men. There were some, the use of which changed the color of the hair, and imparted the gift of memory; while others, less prized, but perhaps equally beneficent in their effects, caused entire oblivion of the past. The voice was improved by some, and made raucous by others. At Cyzicum there was a fountain, called after Cupid, which cured lovers of their passion. Now-a-days, it does not, we believe, require a water of any great strength to produce this effect. Heart affections are measured by the stethoscope and pleximeter, rather than by sentiment and song. Pausanias speaks of a still more wonderful spring, the waters of which renewed virginity. It was annually visited by Juno, with a belief in its possessing this virtue. In the line of marvels, we may mention a spring, without a name, in Ethiopia, of which Herodotus speaks in the following terms: "They who bathed in this fountain gave out an odor of violets, as if they had been thoroughly perfumed, and their bodies were as shining as if they had been rubbed with oil. The water is so light that nothing, even wood, can swim in it. Whatever is thrown into it sinks to the bottom. If this water is truly such as it is represented to be, the continual use of it by the Ethiopians is probably the cause of their longevity, (a hundred years or more)." It was by such narratives as these that Herodotus, the really truthful describer of what he himself saw in his travels, was stigmatized as the father of lies, instead of being universally recognized as "the father of history," a title which is really his due.

From the latter period of the republic to an advanced one of the empire, Baiæ, situated on the western shore of the Bay of Naples, was a fashionable watering-place, and the summer abode of men celebrated in Roman history. Among these may be named Hortensius, Lucullus, Marius, Sylla, Julius Cæsar, Cicero, and Pompey; and among the emperors, Nero, Claudius, Caligula, and Adrian. The Island of Capreæ, the retreat of Tiberius, was close by, although he ended his days at Baiæ by suffocation. Changed is now the scene from that described by Horace, who wrote: There was not to be found in the world a more delightful region than that of Baiæ.* Before long, however, this enchanting spot became the rendezvous of courtezans and even prostitutes, and the moral atmosphere of the place became as tainted and deadly to mental purity as the physical is to the bodily health at the present time. Hot springs furnished water as well as vapor for every variety of thermal bathing, with which could be easily alternated sea-baths. Even at the present day, the traveler, when coasting along the bay in his boat, a privilege which we once enjoyed, sees the substructions jutting out into the sea, of some of the ancient Roman villas, and thermæ, and temples. We need not repeat the encomiums lavished on the spot by Martial, and only make

* Nullus in orbe sinus prælucet amænis. (Lib. i. *Epist.* i.)

passing mention of the adjoining Lucrine lake, celebrated for its green oysters, and once part of the naval depot (*Portus Julius*) of the Romans in this quarter. It communicated in one direction with Lake Avernus, and in another with the sea by a canal.

Fortune-tellers, philters, etc., make up the subjects of the second chapter of Dr. Rouyer's volume. "There existed," he writes, "at Rome, a class of women called *sagæ*, who were engaged in petty traffic, which could only be carried on in a clandestine manner; they were fortune-tellers, and, at the same time, procuresses and perfumers, but if they had a specialty, it was in bringing on abortion. Their name, *sagæ*, served to designate those women who devoted themselves to the practice of midwifery, *sage-femmes*." The chief details respecting the callings of the *sagæ* are furnished by the Latin poets, and especially by Tibullus, Propertius, and Ovid, the triumvirate of love, as they have been called by an old commentator. All that related to love in its physical aspects and to debauchery, came within the functions of the *sagæ*. The most lucrative of their various pursuits was the sale of a crowd of preparations intended for purposes not commonly avowed: as, for instance, to obtain the love of a certain person, by means of philters, properly so called; to secure agreeable dreams; to cause hatred; to inflict suffering on somebody; not to speak of philters to bring on abortion, to prevent conception, etc. Philters which had the property of rendering a person unfit for love, are often referred to by the elegiac poets, who seem to dread, not a little, their power in this way. Many of these fortune-tellers came from Thessaly, which, in this respect, had a reputation in ancient times similar to that enjoyed by Bohemia at the present day, as the reputed home of the gipsies, who are scattered through the different countries of Europe. We shall not follow the author in his account of the composition and alleged effects of different philters, coming under the head of *aphrodisiacs*, nor the fictions introduced by the poets, explaining their origin or confirming their properties. Ovid utters sensible advice on the subject, by exhorting his readers not to rely on such means of excitation. Horace, on the other hand, after denouncing the sorceress Canidia, and her vile incantations and philters, professes to have been himself one of her victims.

The *sagæ* were reputed to be able, by their incantations, to unfit persons *ad militiam veneream*. The Romans made use of two words to designate these juggleries, viz., *præligare*, when it was a question to prevent the first intercourse between two lovers; and *nodum religare*, when the incantation was intended to interrupt an intercourse already established. Ovid speaks of cicuta as an aphrodisiac, and Pliny of the *nymphæa alba*, and of the still more potent *nymphæa heraclea*. The Athenian women, during the Thesmophoria, or festivals in honor of Ceres, in which they took a vow of continence, made large use of the *vitex agnus castus*, called by the Greeks *lygos* or *agnos*, and scattered it over their beds.

Dr. Rouyer terminates this chapter by what will strike many of his readers as a specimen of book-craft. He thinks it apropos of the numerous passages which he has quoted to introduce some biographical notices of their authors, viz., Pliny, Ovid, and Tibullus. Of these, Ovid, if we may judge from the greater space allotted to him, would seem to be the favorite. At the end of the fourth chapter, similar notices of Martial and Juvenal are furnished.

Abortion, as so largely practiced among the Romans, is described in the third chapter of this volume. It was had recourse to from different motives. First and mainly, to prevent exposures from unlawful intercourse, and thus to remove a bar to debauchery; and secondly, to avoid the loss of symmetrical outlines of the body caused by pregnancy, and the diminution of female charms growing out of parturition and the cares of maternity. Ovid says plainly, that women caused themselves to abort in order, by this crime, to prevent the wrinkles and folds of the skin of tne abdomen left by pregnancy. Ovid, not commonly good authority in a question of morals, expresses himself like a sound physiologist, in his pointing out the danger of abortion to the health and even life of the woman, who in attempting to destroy her uterine burden, brings deserved death on herself. The passage is worth transcribing.* "Young girls have recourse to it, but not with impunity; for she who endeavoring to destroy the child in her womb is often the victim of these attempts; she perishes—carried, with her hair disheveled, to her bed, and those around exclaim—She has deserved it!"

The infamous specialty of practicing abortion belonged to the *sagæ*, who were occasionally aided in their criminal manœuvres by the family nurses; these latter, as their young charge grew up to womanhood, became their confidantes and advisers. The Roman law enjoined severe punishments on those who were guilty of procuring abortion; but with the increasing corruption of manners under the emperor, they were seldom enforced. Julia, the niece and concubine of Domitian, who was in the habit of destroying the fruit of her illegitimate love by abortion, finally fell a victim to one of these unnatural and criminal acts.

Dr. Rouyer, by a transition to our own times, describes an unhappily

---

* At teneræ faciunt, sed non impune, puellæ.
Sæpe suos utero quæ necat ipsa perit;
Ipsa perit, ferturque toro resoluta capillos;
Et clamant: "Merito!" qui modo cumque vident.
(*Amores*, lib. ii el xiv.)

too close imitation of the manners and vices of ancient Rome in modern Paris, in which city there are houses ostensibly for the reception of women during their confinement, (*maisons soi-disant d'accouchements,*) but in reality open asylums for prostitution and the procuring of abortion, some of which are even known to strangers. The law is often at fault in exposing and punishing the chief actors in these vile receptacles, owing to the want of physical proofs, although those of a moral nature might be deemed conclusive. When a sufferer from the manœuvres to bring on abortion, alarmed for her life, consults a physician, he feels, naturally, indignation at the atrocity of the act, but he is restrained from at once ferreting out and denouncing the criminal by his implied obligations to preserve the secret of his patient.

Eunuchs, the theme of the fourth chapter of the volume before us, furnish a still more atrocious example than any of the preceding ones which have been brought to our notice by the author, of the cruelty and licentiousness of the inhabitants of imperial Rome. The *lex talionis* invoked in the malediction pronounced by Ovid, that "he who the first deprived children of their genital organs ought himself to have been subjected to the operation," could not have been carried into effect, if we put faith in the assertion of both Claudian and Ammianus Marcellinus, that Semiramis, the famed queen of Assyria, the wife and successor of Ninus, was the first "*quæ teneros mares castravit.*" The author distinguishes the different kinds of castration resorted to with a view of making eunuchs, who were designated accordingly. The *castrati* were those from whom all the external organs of generation had been removed; the *spadones* had lost their testicles; and a third class, *thlibiæ*, retained their virile organs, but their testicles had been crushed and the procreative functions in consequence abolished. Sometimes these last were designated by the term *thlasiæ*. The first were the most prized, and cost much more than the others—a difference which is found to prevail in the Eastern world in which this vile practice is still kept up. Of its great antiquity in those countries, an idea may be formed from the fact that in the times of ancient Rome a great many eunuchs came from Persia. The Island of Delos was also celebrated for its depot of eunuchs. Castration was practiced by barbers, (*tonsores,*) and also by the *mangones*, those who were more especially engaged in the trade of these imperfect specimens of humanity. The operation was usually performed at a very tender age, or soon after birth, but sometimes after the period of puberty.

Eunuchs were in great demand among the Romans, and particularly among the Roman women *ad securas libidinationes*, says St. Jerome.*

* These degraded creatures were sometimes used for still viler purposes, and played a double part: *inter fœminas viri, et inter viros fœminæ.*

Domitian, notwithstanding all his cruelties and tyranny, issued an edict against the process of emasculation, which was confirmed by his successor Nerva. On the other hand, Heliogabalus was liberal in his gifts of money and of office to eunuchs. The severe moralist will perhaps look with a more lenient eye on castration when practiced as a punishment for adultery, as in the cases of Carbo Attienus and Marcus Pontius, who were caught *in flagrante delictu.* Martial twits a poor husband for threatening to cut off the nose of his wife's lover, with the taunting remark, that "this was not the offending organ. Poor fool!"

A few lines are given by Dr. Rouyer to the unsexing of women, which was first practiced by Gyges, King of Lydia, in order that his wives might preserve for a longer period their youthful freshness and beauty. It is most probable that the operations referred to by the ancient writers were not excision of the uterus or ovaries, but resection of the clitoris when too largely developed, or of hypertrophied *labiæ minoræ.* This last operation is still practiced in certain countries.

A short chapter (v.) is given to the practice of infibulation in ancient Rome. It is described by Celsus (lib. vii. xxv.,) who assigns as reasons for performing it, the preservation of the voice and regard for health, *valetudinis causâ.* Infibulation by a silver ring through the prepuce was practiced to prevent too early sexual intercourse. At a more advanced age the fibula was removed, and this event was coincident with the assuming of the toga, as an evidence of manhood and its privileges. Actors and singers were subjected to infibulation by the person who had charge of the theatres. Physiologically, one cannot see how the voice would be improved by this process, as in the cases of castration, except by preventing venereal excesses, and thus withholding one cause of debility and languor, which would interfere with the pitch and clearness of vocalization.

Connected with the history of the *fibula,* is that of the *subligar* or *subligaculum.* It was a kind of leathern apron which covered the genital organs and extended from the waist to the knees. All actors when on the stage were required to wear the subligar. Its use was equally enjoined on those who entered the bath. The reader must bear in mind that neither pantaloons nor drawers formed any part of the dress of an inhabitant of ancient Rome.

Cosmetics and perfumes were in great demand among the nations of antiquity; and even the physicians did not think it beneath them to direct their attention to the various practices in use for embellishing the body. Numerous formulas of preparations for the purpose are to be found in Hippocrates, Celsus, Galen, Paul of Egina, etc. The objects to be attained were twofold: All that related to hygiene and beautifying the body came under the *ars ornatrix,* or *cosmetic;* carrying things still further, the *ars fucatrix* or *commotic* was reached. This latter was the art of arresting natural imperfections and of repairing the ravages of time. The Romans carried the use of perfumes to an excess, as they did every luxurious enjoyment. No festival or symposium was complete without aids of this kind. Their baths were perfumed, as were the theatres and their chambers and beds. Their most esteemed wines received a *bouquet* from the same source. Odoriferous substances were put on the funereal pyre and burnt with the body. Martial apostrophizes a certain Zoilus as a vile rogue, and charges him to "restore speedily those perfumes, that cannella, that myrrh which still exhales a funereal odor, that incense abstracted from the flames of the pyre, and that cinnamon which you have stolen from the bed of death."*

Egypt, Arabia, and India were put under contribution for obtaining the substances out of which the perfumes were afterwards manufactured in Rome. Italy itself furnished for this purpose the lily, the iris, the oenanthe, the narcissus, the marjolan, etc. The roses of Pæstum enjoyed at that time a great reputation, and were celebrated by the poets both for their delightful scent and their twice yearly flowering—*biferi rosaria Pæsti.* The odorous reed (*andropogon schœnanthus*) furnished also one of the commonest and the least esteemed perfumes: it was almost exclusively used by the frail sisterhood, who on that account were sometimes called *schoeniculæ*, from *schænus*, the received name of the plant. This class of women were forbidden to make use of the choicer perfumes, as they were to wear certain stuffs and dresses, the use of which was reserved for matrons. They were also forbidden to wear black hair, and were still more degraded by being required to dye their own of a yellow or blue color.

The *ars fucatrix*, or *commotic*, opens to our notice questions of a more varied and interesting nature. The trade in it included a certain number of specialties which are practiced even at the present time. The philocommetic preparations were extremely numerous among the Romans. Pliny is quite liberal in giving the formulas for many of them. The pear enjoyed a high reputation for its many properties: it was thought to be aphrodisiac, to improve the voice and to favor sleep; and the peelings, when boiled, served to dye the hair of a black color. Bear's grease was as much extolled for its alleged hair-growing virtues as it was recently in our own day. The Latin poets are profuse in their references to and

---

* Unguenta et casias et olentem funera myrrham
Thuraque de medio semicremata rogo,
Et quæ de Stygio rapuisti cinnama lecto;
Improbe, de turpi redde, Zoile, sinu. —Lib. xi. ep. 55.

descriptions of the cosmetic devices adopted by the women. This was still more the case in reference to the preparations destined to preserve the freshness of the complexion and the softness of the skin. The empress Poppæa always kept near her, in Rome, and took with her on her journeys, a large number of asses, whose milk supplied her baths. The most celebrated soap came from Gaul: it was of two kinds, the soft and the liquid, and was said to be made of goat's fat and the ashes of the beech.

The chief pigments used for imparting a white color to the skin were white-lead and chalk; while the red was procured from carmine and a substance taken from the excrements of the crocodile, and also, according to Pliny, bull's dung. Ovid is still more minute in revealing the mysteries of the toilet by which the Roman ladies endeavored to repair the ravages of time—*munditiis annorum damna rependunt.* Addressing one of them, the poet says: "You borrow the deceitful whiteness of ceruse; other artifices supply the color of the blood. You know how to stretch or to render thicker your eyebrows, and to conceal your real cheeks under a cosmetic. You are not ashamed to give additional lustre to your eyes by the use of fine powders, or by saffron, which grows on the banks of the limpid Cydnus."* Ovid composed a poem entitled *Cosmetics*, (*Medicamina Faciei,*) which he dedicated to the other sex, as we see in the first two verses:—

Discite quæ faciem commendat cura, puellæ,
Et quo sit vobis forma tuenda modo.

The greater part of this work is lost, but a fragment of a hundred verses remaining, bears evidence of the astonishing faculty in versifying, by which he could give, in harmonious numbers, eight different articles, and the proportion by weight of each of them, for a formula, intended to give a uniform and more brilliant hue to the skin. Martial often speaks of women who make excessive use of ceruse and chalk, in these lines:—

Sic, quæ nigrior est cadente moro
Cerussata sibi placet Lycoris.—Lib. ix. ep. 38.

"Lycoris, who is blacker than a ripe mulberry, thinks herself beautiful when she is whitened with ceruse."

We learn from many epigrams of this poet that dental prothesis was in high repute among the Romans. "Thais has black teeth, Lecania has white ones. Why is this? The former has her own teeth, the latter purchased ones."† The testimony of Martial refers only to the dentistry

---

* Ars Amoris III.

† Theus habet nigros, niveos Lecanea dentes;

Quæ ratio est? Emptos hæc habet, illa suos.

(Lib. v. ep. 43 )

of the first century of our era; but there are proofs of an earlier period of this art in the tenth of the Laws of the Twelve Tables, which date as early as 450 years before Christ. To the prohibition to bury gold with the dead an exception was made in the case of that which was found in the mouth of the deceased for the purpose of fastening the teeth together.

Depilation was carried to a great extent in the luxurious and degenerate period of Rome. All parts of the body were, for various reasons, subjected to the operation—the face, forehead, armpits, arms, hands, legs. Cicero speaks of depilation of the eyebrows; but it was most practiced in the region of the genital organs, and to the greatest possible extent. Women took the lead in these operations, which were executed, in the first instance, probably from considerations of cleanliness and of hygiene, but afterwards from the requirements of perverse fashion. Pliny mentions a number of depilatories. The preparations specially employed for the purpose were called *psilothrum* and *dropax,* also friction with pumice-stone. In some cases the hair was pulled out by tweezers named *volsellæ;* and in others the parts were shaved with a rasor, (*novacula.*)

We find that our detached notices of Dr. Rouyer's volume have already led us farther than we intended, and that we must, therefore, postpone to another time an account of the latter portion of it, which consists of an historical sketch of women engaged in midwifery in ancient times, and thence, by not very well marked gradations, through the middle ages down to those of our own day—or from the goddess Juno to Madame Boivin and Doctress Elizabeth Blackwell. A separation may well be made between the subjects which we have brought under the eyes of our readers and the contents of the last chapter on "Medical Studies," etc.

